# Orexin‐A Attenuates the Inflammatory Response in Sepsis‐Associated Encephalopathy by Modulating Oxidative Stress and Inhibiting the ERK/NF‐κB Signaling Pathway in Microglia and Astrocytes

**DOI:** 10.1111/cns.70096

**Published:** 2024-11-07

**Authors:** Jing Guo, Dexun Kong, Junchi Luo, Tao Xiong, Fang Wang, Mei Deng, Zhuo Kong, Sha Yang, Jingjing Da, Chaofei Chen, Jinhai Lan, Liangzhao Chu, Guoqiang Han, Jian Liu, Ying Tan, Jiqin Zhang

**Affiliations:** ^1^ GuiZhou University Medical College Guiyang Guizhou China; ^2^ Guizhou Medical University Guiyang China; ^3^ Department of Neurosurgery Guizhou Provincial People's Hospital Guiyang China; ^4^ Department of Nephrology Guizhou Provincial People's Hospital Guiyang China; ^5^ Institute of Pediatrics, Guangzhou Women and Children's Medical Center Guangzhou Medical University Guangzhou China; ^6^ Department of the Second Surgery Ziyun People's Hospital Anshun China; ^7^ Department of Neurosurgery The Affiliated Hospital of Guizhou Medical University Guiyang China; ^8^ Department of Anesthesiology Guizhou Provincial People's Hospital Guiyang China

**Keywords:** astrocytes, microglia, neuroinflammation, oxidative stress, sepsis‐associated encephalopathy

## Abstract

**Background:**

Oxidative stress‐induced inflammation is a major pathogenic mechanism in sepsis‐associated encephalopathy (SAE). We hypothesized that regulation of reactive oxygen species (ROS) by the neuropeptide orexin‐A could prevent SAE‐induced oxidative stress and inflammation. Therefore, the aim of this study was to investigate the effects of orexin‐A on oxidative stress and inflammation in SAE in mice.

**Methods:**

Adult male mice were treated with orexin‐A (250 μg/kg, intranasal administration) to establish a cecal ligation perforation (CLP) model. We performed behavioral tests, observed neuronal damage in the hippocampal region, measured the levels of ROS, NOX2, and observed the structure of mitochondria by transmission electron microscopy. We then examined the inflammatory factors TNF‐α and IL‐1β, the activation of microglia and astrocytes, the expression of ERK/NF‐κB, C3, and S100A10, and the presence of A1 type astrocytes and A2 type astrocytes.

**Results:**

Orexin‐A treatment improved cognitive performance in CLP‐induced SAE mice, attenuated neuronal apoptosis in the hippocampal region, ameliorated ROS levels and the extent of mitochondrial damage, and reduced protein expression of NOX2 in hippocampal tissue. In addition, orexin‐A treatment significantly reduced microglia and astrocyte activation, inhibited the levels of P‐ERK and NF‐κB, and reduced the release of IL‐1β and TNF‐α, which were significantly increased after CLP. Finally, Orexin‐A treatment significantly decreased the number of C3/glial fibrillary acidic protein (GFAP)‐positive cells and increased the number of S100A10/GFAP‐positive cells.

**Conclusion:**

Our data suggest that orexin‐A reduces ROS expression by inhibiting CLP‐induced NOX2 production, thereby attenuating mitochondrial damage and neuronal apoptosis. Its inhibition of microglial and A1‐type astrocyte activation and inflammation was associated with the ERK/NF‐κB pathway. These suggest that orexin‐A may reduce cognitive impairment in SAE by reducing oxidative stress‐induced inflammation.

## Graphical abstract

This graphic abstract provides an overview of the paper, focusing on the neuromodulatory effects of the neuropeptide orexin‐A (OXA) in sepsis‐associated encephalopathy. OXA reduces reactive oxygen species‐induced oxidative stress by inhibiting NOX2 expression in hippocampal tissue. The main mechanism may be by inhibiting the activation of A1‐type astrocytes and microglia with the NF‐κB/ERK signaling pathway, which reduces the expression of pro‐inflammatory factors TNF‐α and IL‐1β and reduces neuronal apoptosis.

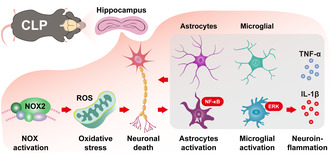

## 1 Introduction

Sepsis is a systemic inflammatory response induced by infection and tissue damage and is a major focus of modern medical research. Progression of sepsis can lead to severe sepsis, septic shock, and a clinical mortality rate of up to 80% [1]. Among these, sepsis‐associated encephalopathy (SAE) is one of the most common complications, which is a diffuse cerebral dysfunction due to a systemic inflammatory response with severe neurological damage, with varying degrees of impaired consciousness, including hallucinations and delirium, and in severe cases, deep coma or even death [2]. Neurological damage occurs in patients who survive the acute and post‐acute phases of sepsis [3]. Therefore, there is an urgent need to explore its pathogenesis in depth and to find effective drugs to reduce the morbidity and the severity of infectious brain damage.

Oxidative stress damage is one of the key mechanisms in the pathogenesis of SAE [4]. The pathogenesis of septic encephalopathy caused by reactive oxygen species (ROS) has received much attention in recent years [5]. Research has shown that ROS are a determinant of cognitive dysfunction after sepsis [6]. The excessive production of ROS induces oxidative stress, which results in oxidative damage to cells, leading to inflammation and ultimately apoptosis, which is an important basis for concurrent brain dysfunction [7, 8]. Therefore, how to maintain ROS homeostasis and regulate oxidative stress‐induced inflammatory damage may be the focus of SAE treatment.

Orexin‐A (OXA) is a peptide secreted by the hypothalamus that is involved in a variety of physiopathological processes in organisms, including sleep‐wakefulness, reward‐seeking, and cardiovascular responses [9]. Recent studies have shown that OXA attenuates inflammatory responses and has neuroprotective effects [10]. Notably, previous studies have shown that OXA reduces ROS overproduction by attenuating oxidative stress, thereby protecting against the occurrence of neuronal death [11], ischemic brain injury [12], and hepatocyte inflammation [13]. Therefore, OXA could be a potential therapeutic agent for modulating oxidative stress injury diseases. However, the therapeutic effect of OXA on ROS‐induced oxidative stress after septic brain injury is unknown.

Astrocytes, which are the most widely distributed in the brain, and microglia have a variety of functions, such as the brain's innate immune response [14]. Studies have indicated that inhibition of microglia and astrocyte overactivation attenuates the occurrence of septic brain injury [15]. Strong interaction between microglia and astrocytes is a key factor in the progression and severity of neurological disease [16]. Extracellular signal‐regulated kinase (ERK) and nuclear factor‐κB (NF‐κB) are considered key molecules leading to microglial and astrocyte activation after sepsis [17, 18]. These findings suggest the importance of regulating microglial and astrocyte activation in the treatment of septic encephalopathy. However, although previous studies have found that OXA attenuates the inflammatory response, whether OXA affects the inflammatory response induced by microglia and astrocyte activation after the onset of sepsis and the possible mechanisms are unknown.

Therefore, the main aim of our study was to evaluate the antioxidant and anti‐inflammatory effects of OXA on the hippocampal region of mice with CLP‐induced SAE and to elucidate the potential mechanisms of action.

## 2 Methods

### 2.1 Animals

The experimental animals were healthy SPF grade C57BL/6J male mice, 6–8 weeks old, weighing 20–24 g, provided by Chongqing Tengxin Biotechnology Company. They were fed for 1 week under standard feeding conditions at room temperature of 20°C–24°C and natural day and night lighting. This experiment complied with the regulations of Guizhou Provincial People's Hospital and Guizhou Provincial Animal Protection Institution regarding the feeding of experimental animals. It conformed to the requirements of animal ethics and followed the basic principle of minimizing harm to the subjects.

### 2.2 CLP‐Induced Septic Mouse Model

The mouse sepsis model was established by cecal ligation and perforation (CLP) as previously described [19, 20]. Briefly, after 12 h of preoperative fasting, sevoflurane inhalation anesthesia was applied, and then the mice were placed on a surgical plate and the limbs were fixed. Under aseptic conditions, the cecum was cut approximately 1.0–1.5 cm along the midline of the abdomen to expose the cecum but not to damage the mesenteric vessels and ligated with a 5–0 silk ligature. A 22G syringe‐puncturing needle was used to puncture from one side of the cecum to the other, and a small amount of feces was squeezed gently and extruded from the cecum. The cecum was returned to the abdominal cavity, and the incision was then closed layer by layer. The sham group underwent the same operation but without ligation and puncture. Postoperatively, the surgical site was coated with iodophor, and all animals were injected subcutaneously with saline (5 mL/100 g) to replace the water lost during the surgery.

### 2.3 Drug Treatment

OXA was purchased from MedChemExpress and dissolved in saline. Intranasal administration of 250 μg/kg of OXA drug [21], 2.5 μL per nostril, and 5.0 μL per mouse was used in this experiment.

### 2.4 Experimental Design

#### 2.4.1 Experiment 1. To Test the Effects of Orexin‐A on Cognitive and Behavioral Changes in Septic Mice

The mice were randomly divided into 4 groups: Sham group (Sham), Sham + orexin‐A 250 μg/kg group (Sham + OXA), CLP group (CLP), and CLP + orexin‐A 250 μg/kg group (CLP + OXA group). The CLP method was used to establish the sepsis mouse model. OXA was administered intranasally once a day for 7 days, and the behavioral observation of the surviving mice was performed 7 days after the surgery (12 in each group).

#### 2.4.2 Experiment 2. To Observe the Effects of OXA on Neuronal Apoptosis and Oxidative Stress Damage in the Hippocampal Region of Septic Mice

The animals were grouped as in experiment 1. At the end of the behavioral experiments, the brains of the executed mice were taken and frozen sections were performed. TUNEL and NeuN staining were used to assess CLP‐induced damage and survival of neurons in the CA1 region of the hippocampus in SAE (3 in each group).

Hippocampal tissue ROS content was detected, and mitochondrial damage was observed using transmission electron microscopy (3 in each group). Determine the protein expression level of NOX2 in the hippocampal tissue of each group of mice (3 in each group).

#### 2.4.3 Experiment 3. To Study the Effect of OXA on Inflammatory Factors in Septic Mice

As in the group of experiment 1, the brains of the mice were removed on day 7 after surgery, serum was collected, and the hippocampus was dissected. Serum levels of inflammatory factors (IL‐1β, TNF‐α) were determined by ELISA in each group of mice (6 in each group). In another group of mice, the protein expression levels of IL‐1β and TNF‐α in hippocampal tissues were detected by Western blot (3 in each group).

#### 2.4.4 Experiment 4. To Study the Effect of OXA on the Activation of Microglia and Astrocytes and Inflammation‐Related Signaling Pathways

Grouped as in experiment 1, microglia were labeled with Iba‐1 and astrocytes were labeled with GFAP, and the activation was observed by immunofluorescence (3 in each group).

In the exploration of mechanisms, the protein expression levels of Iba‐1, GFAP, P‐ERK, and NF‐κB in mouse hippocampal tissues were detected (3 in each group). In another group of mice, immunofluorescence staining was performed to detect the co‐expression of P‐ERK and NF‐κB in microglia and astrocytes, respectively (3 in each group).

### 2.5 Behavioral Testing

#### 2.5.1 Open‐Field Test (OFT)

The OFT is a classical behavioral test used to assess spontaneous activity, anxiety, and exploratory behavior in animals [22]. The experimental setup is a white opaque cube with a box size of 50 cm × 50 cm × 40 cm. During the experiment, the mice were gently placed in one corner of the box and allowed to move freely in the open field for 5 min, which was automatically recorded using a video tracking system (RWD Life Technologies Ltd) to analyze the distance moved and the time spent in the central area during the 5 min. At the end of the test, each animal was wiped with 75% alcohol to remove odor.

#### 2.5.2 Y‐Maze

The Y‐maze experiment is mainly used to test the learning memory ability of animals [23]. The Y‐maze consists of 3 arms; the angle between the arms is 120°, and the size of each arm (*L* × *W* × *H*) is 10 cm × 5 cm × 5 cm. During the training process, the mice were placed in the Y‐maze to explore freely for 10 min by closing the novel arm with a block. After the training was completed, the mice were placed back in the cage to rest for 1 h. The flaps of the neo‐alien arm were removed after the training process, and the mice were allowed to enter the new arm and move freely in the three arms, and the residence time and distance of the new arm within 5 min were recorded. %time = (novel arm activity time/test time) × 100%. %travel distance = (novel arm distance/total distance) × 100%.

#### 2.5.3 Tail Suspension Test

The tail suspension test is used to assess the effects on depression‐related behaviors [24]. In a dark, quiet room, mice were taped approximately 1 cm from the end of their tails and hung upside down with their heads approximately 30 cm above a tabletop, and the resting time of the mice was accumulated over 5 min.

### 2.6 TUNEL Staining to Detect Changes in Neuronal Apoptosis

Sections were rinsed in 0.1 M PBS and incubated in 0.1% Triton X‐100 for 30 min, followed by sealing with 5% goat serum for 1 h. rTdT incubation buffer was configured according to the instructions of the kit (E‐CK‐A320, Elabscience), and incubation was carried out in light‐protected incubation for 1 h at 37°C. After 3 washes in PBS, DAPI was added. Sections were sealed, and images were captured using laser confocal (Zeiss, LSM 980).

### 2.7 Determination of Tissue ROS

ROS levels in hippocampal tissue were detected with a fluorescent probe following the protocol described in the kit (BB‐470523, Bestibio). Samples were imaged using a fluorescence microscope (Zeiss, LSM 980).

### 2.8 Enzyme‐Linked Immunosorbent Assay (ELISA)

Inflammatory cytokines were detected using commercially available ELISA kits (mouse TNF‐α, EK0527, Boster); (mouse IL‐1β, EK0394, Boster) and the optical density at 450 nm was analyzed spectrophotometrically.

### 2.9 Western Blotting

Hippocampal tissue was extracted immediately after mouse execution, and protein concentration was determined by BCA. Equal amounts of protein (30 μg) were separated on a 10% acrylamide gel and transferred to a PVDF membrane. Primary antibodies configured with antibody dilutions were added after sealing with rapid sealing solution. Primary antibodies included: rabbit anti‐P‐ERK (#9926, Cell Signaling Technology), rabbit anti‐ERK (#9926, Cell Signaling Technology), rabbit anti‐NOX2 (SN07‐16, HuaBio), rabbit anti‐TNF‐α (A11534, Abclonal), rabbit anti‐NF‐κB (SZ10‐04, HuaBio), rabbit anti‐IL‐1β (#31202, Cell Signaling Technology), mouse anti‐Iba‐1 (GB123502‐100, Servicebio), mouse anti‐GFAP (#3670, Cell Signaling Technology), −4°C incubation overnight. β‐actin (GB15003‐100, Servicebio) was used as an internal reference. After three washes with TBST, horseradish peroxidase (HRP)‐linked secondary antibody (GB23303, Servicebio) was added and washed three times at room temperature for chemiluminescence development, and the results were analyzed using ImageJ software.

### 2.10 Immunofluorescence

Mouse brain tissue was cryosectioned and incubated with 10℅ goat serum (G1208, Servicebio) for 1 h at room temperature and then incubated with primary antibody at 4°C overnight. The primary antibodies used were: mouse anti‐Iba‐1 (GB123502‐100, Servicebio), mouse anti‐GFAP (#3670, Cell Signaling Technology), rabbit anti‐Iba‐1 (EPR16588, Abcam), rabbit anti‐P‐ERK (#9926, Cell Signaling Technology), rabbit anti‐NF‐κB (SZ10‐04, HuaBio), rabbit anti‐C3 (ET1702‐99, HuaBio), rabbit anti‐S100A10 (ET1702‐38, HuaBio), and rabbit anti‐Neun (#24307, Cell Signaling Technology). After three washes with PBS, the sections were incubated with coupled anti‐rabbit and anti‐mouse secondary antibodies Alexa Fluor Plus 594 (A‐11062, Invitrogen), Alexa‐488 (A‐32731, Invitrogen), and Alexa Fluor Plus 594 (A‐11005, Invitrogen), Alexa‐488 (A‐32723, Invitrogen) at room temperature for 1 h. After rinsing in PBS, the sections were incubated with DAPI (G1407, Servicebio) to stain the sections. Final observation was performed using a laser scanning confocal microscope (Zeiss, LSM 980) and analyzed using the ImageJ software.

### 2.11 Statistical Analysis

Data were processed and analyzed using GraphPad Prism 8.0 software. All measurements were expressed as mean ± standard deviation (X ± SEM). Comparisons between two or more groups were made using one‐way ANOVA followed by Tukey's post hoc test. *p* < 0.05 was considered statistically significant.

## 3 Results

### 3.1 OXA Treatment Attenuates Cognitive Dysfunction in SAE Mice

To verify the presence of significant cognitive dysfunction in septic mice after CLP surgery at 7 days post‐operatively and to assess the therapeutic effect of OXA on sepsis‐induced cognitive dysfunction, we used behavioral tests that reflect the animals' ability to learn and remember.

**FIGURE 1:**
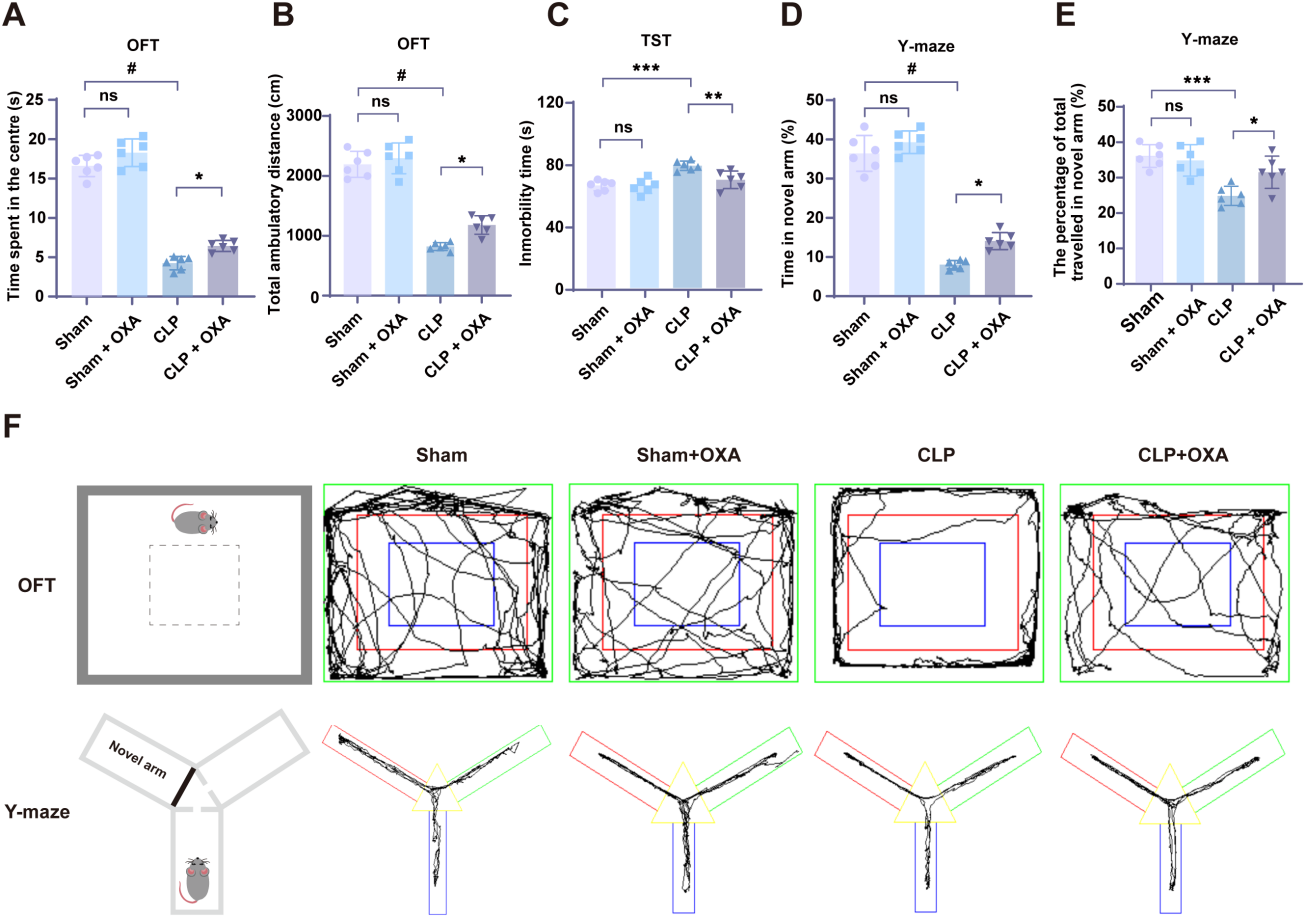
OXA improves cognitive impairment in septic mice. Behavioral tests were performed seven days after CLP. (A, B) Time spent in the center of the open field test versus total distance moved. (C) Time at rest during 5 min of the tail suspension test. (D, E) Percentage of time spent in the novel arm of the Y‐maze and percentage of distance traveled. (F) Activity trajectories of representative open field test, and Y‐maze. One‐way ANOVA and Tukey's post hoc test were used. The values were presented as mean ± SD (*p < 0.05, **p < 0.01, ***p < 0.001, ^#^ *p* < 0.0001, *n* = 6 for each group).

On the seventh day after the administration of OXA, surviving mice were subjected to the OFT, the Y‐maze test, and the TST, respectively. As shown in Figure 1A,B, in the OFT used to assess spontaneous activity, anxiety state, and exploratory behavior of the animals, the difference between the Sham and the Sham + OXA groups of mice in terms of central dwell time and total distance of activity was not statistically significant. Compared with the Sham group, mice in the CLP group showed a significant decrease in central residence time and total activity distance (^#^ *p* < 0.0001, ^#^ *p* < 0.0001), whereas administration of OXA resulted in a significant increase in central residence time and total activity distance (*p < 0.05, *p < 0.05 vs. CLP group).

As shown in Figure 1C, in the TST used to assess anxiety status, there was no significant difference in the total resting time over 5 min between the Sham and Sham + OXA groups of mice. Compared to the Sham group, the CLP group showed a significant increase in the resting time within 5 min (***p < 0.001 vs. Sham group) and a decrease in the total resting time within 5 min in the CLP + OXA drug group (**p < 0.01 vs. CLP group).

As shown in Figure 1D,E, there was no significant difference between Sham group and Sham + OXA group mice in terms of neo‐arm dwell time and active distance in the Y‐maze used to assess exploratory behavior. The novel arm dwell time and activity distance were significantly lower in the CLP group compared with the Sham group (^#^ *p* < 0.0001, ***p < 0.001). Novel arm dwell time and activity distance were significantly increased in the CLP + OXA drug group (*p < 0.05, *p < 0.05 vs. CLP group).

These results indicated that significant septic brain damage occurred after CLP, resulting in a decrease in basic motor activities, the emergence of anxiety‐like behaviors, and deficits in exploratory skills. However, intranasal OXA administration for 7 consecutive days effectively reduced CLP‐induced cognitive and anxiety deficits and improved exploratory abilities in septic mice.

### 3.2 OXA Attenuates Neuronal Apoptosis in the Hippocampus of SAE Mice

**FIGURE 2:**
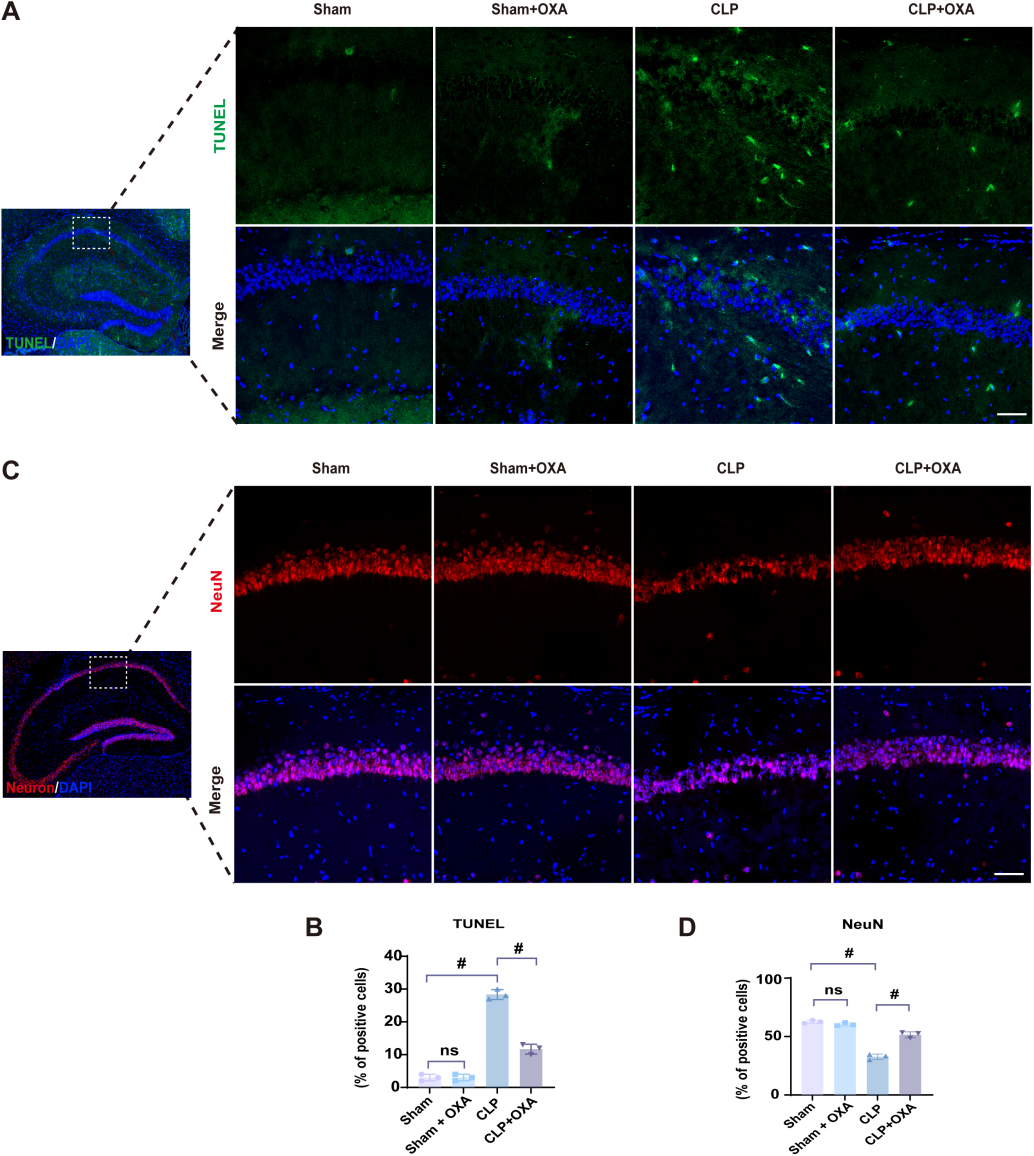
OXA treatment attenuates hippocampal neuronal damage after CLP. (A, B) TUNEL staining showing neuronal apoptosis and quantification of TUNEL (green) with DAPI (blue) in the CA1 area of the hippocampus 7 days after CLP. Scale bar = 50 μm. (C, D) NeuN staining showing neuronal survival in the hippocampal CA1 area 7 days after CLP and corresponding statistical analysis. NeuN+ (red) and nuclei (blue). Scale bar = 50 μm. One‐way ANOVA and Tukey's post hoc test were used. The values were presented as mean ± SD (^#^ *p* < 0.0001, *n* = 3 for each group).

Neuronal apoptosis in the hippocampus is a major cause of cognitive impairment [25]. TUNEL and NeuN staining were used to assess the apoptosis and survival of neurons in the CA1 region of the hippocampus after CLP injury (Figure 2). As shown in Figure 2A,B, TUNEL‐stained positive cells represented apoptotic neurons. The Sham and Sham+OXA groups had less apoptotic neuronal staining, and the difference was not statistically significant. The number of apoptotic neurons in hippocampal tissue was significantly increased in the CLP group (^#^ *p* < 0.0001 vs. Sham group), whereas OXA treatment significantly decreased the number of apoptotic neurons in the CA1 region of the hippocampus (^#^ *p* < 0.0001 vs. CLP group).

NeuN‐staining positive cells responded to surviving neurons. As shown in Figure 2C,D, the neurons in the hippocampal CA1 region were densely arranged and clearly hierarchical in the Sham group and the Sham + OXA group, and the difference was not statistically significant. The number of surviving neurons in the hippocampal CA1 region was significantly lower in the CLP group (^#^ *p* < 0.0001 vs. Sham group), and the proportion of NeuN‐positive neurons was significantly increased by OXA treatment (^#^ *p* < 0.0001 vs. CLP group). The above results suggest that OXA treatment reduces CLP‐induced neuronal apoptosis in the hippocampal CA1 region of SAE mice.

### 3.3 OXA Inhibits Oxidative Stress Injury to the Hippocampus in SAE Mice by Reducing NOX2 Expression

**FIGURE 3:**
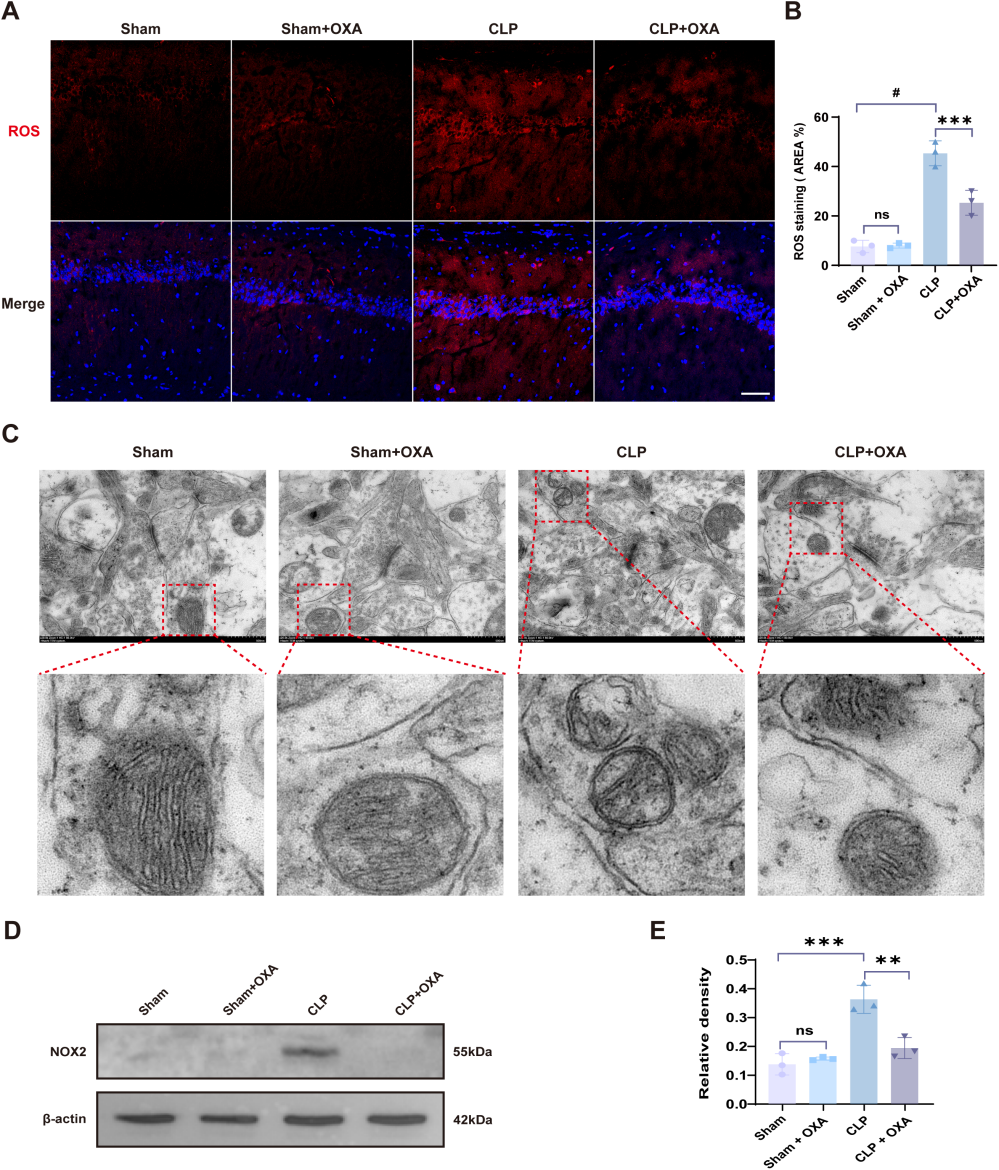
Inhibition of NOX2 expression by OXA reduces ROS generation to decrease oxidative stress damage. (A, B) Representative images and quantification of ROS immunofluorescence staining in the hippocampal region. Scale bar = 50 μm. (C) Representative images of synaptic mitochondrial ultrastructure and enlarged mitochondrial ultrastructure in each group. Scale bar = 500 nm. (D, E) Protein expression and relative quantification of NOX2 in hippocampal tissue. One‐way ANOVA and Tukey's post hoc test were used. The values were presented as mean ± SD (**p < 0.01, ***p < 0.001, ^#^ *p* < 0.0001, *n* = 3 for each group).

Excess ROS production induces neuronal apoptosis, and ultimately protected SAE mice [26]. OXA has been reported as a potential antioxidant. Therefore, we explored the effects of OXA on SAE‐induced oxidative stress injury. First, we examined the expression levels of ROS in neurons in the hippocampus. We observed lower ROS levels in the hippocampal region in the Sham group versus the Sham + OXA group, and the difference was not statistically significant. ROS levels in the hippocampal region were significantly higher in the CLP group (^#^ *p* < 0.0001 vs. Sham group), whereas the ROS levels were reduced after OXA administration (***p < 0.001 vs. CLP group) (Figure 3A,B).

ROS exposure caused mitochondrial damage. We sampled the hippocampal tissue 7 days after surgery and observed the structure of mitochondria by transmission electron microscopy. As shown in Figure 3C, in the Sham and Sham + OXA groups, the inner mitochondrial membrane showed a clear cristae‐like structure, the mitochondrial morphology in the CLP group showed an abnormal structure with a shallow matrix, shorter and fewer cristae, or even disappeared, as well as disruption of the structural integrity of the mitochondrial membrane, and the mitochondrial structure was restored in the CLP + OXA group. OXA has a protective effect against mitochondrial damage after SAE.

**FIGURE 4:**
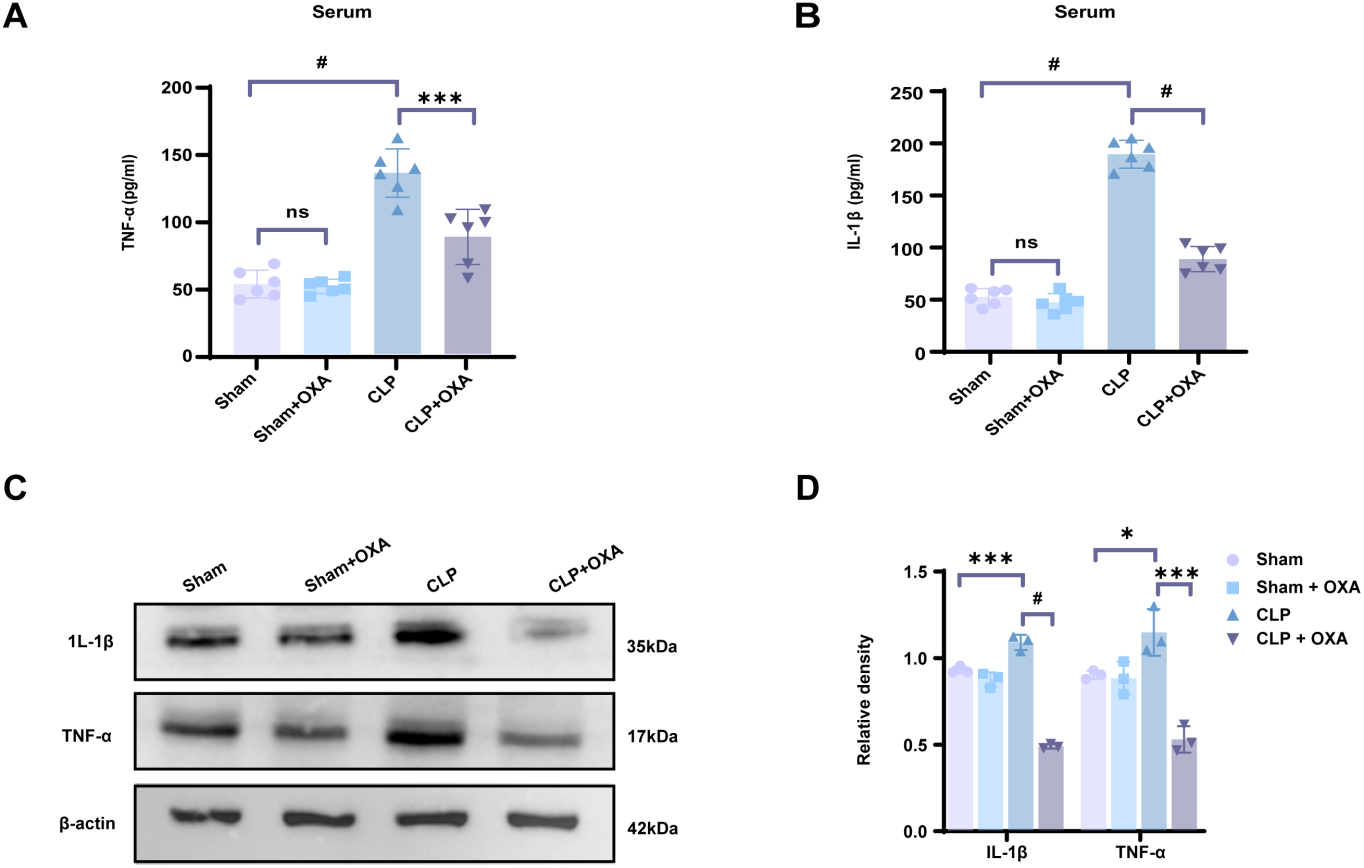
OXA reduces CLP‐induced levels of inflammatory factors. Following the model, OXA was administered intranasally once a day for seven days. After seven days, blood was collected and hippocampal tissue was extracted. Serum (A) IL‐1β and (B) TNF‐α levels in each group. (C, D) Protein expression and relative quantification of IL‐1β and TNF‐α in hippocampal tissue. One‐way ANOVA and Tukey post hoc tests were used. The values were presented as mean ± SD (*p < 0.05, ***p < 0.001, ^#^ *p* < 0.0001, *n* = 6 or 3 for each group).

Further, since one of the major sources of ROS in cells is NOX2, to assess the exact mechanism by which OXA prevents oxidative stress, we evaluated whether OXA could reduce ROS production by inhibiting NOX2 levels. As expected, protein expression of NOX2 was low in hippocampal tissues of mice in the Sham and the Sham + OXA groups, and the difference was not statistically significant. Hippocampal tissue NOX2 protein expression was significantly elevated after CLP (***p < 0.001 vs. Sham group), but NOX2 expression was significantly lower in the CLP + OXA treatment group (**p < 0.01 vs. CLP group) (Figure 4D,E). Taken together, we found that OXA inhibited CLP‐induced oxidative stress injury by suppressing NOX2 production.

### 3.4 OXA Therapy Reduces CLP‐Induced Levels of Inflammatory Factors

Oxidative stress activates the inflammatory response, and the release of pro‐inflammatory factors TNF‐α and IL‐1β causes alterations in sepsis‐induced cognitive abilities [27]. To further explore the effect of OXA on oxidative stress injury‐induced inflammation, we examined the inflammatory factors in the serum of each group 7 days after the model. As shown in Figure 4A,B, the pro‐inflammatory factor levels were lower in the Sham and the Sham + OXA groups, and the difference was not statistically significant. A significant increase in TNF‐α and IL‐1β expression was observed in the CLP group (^#^ *p* < 0.0001, ^#^ *p* < 0.0001 vs. Sham group), and TNF‐α and IL‐1β were reduced in the serum of mice in the CLP + OXA group (***p < 0.001, ^#^ *p* < 0.0001 vs. CLP group).

In addition, we extracted hippocampal tissues to examine protein expression, and as shown in Figure 4C,D, the expression of TNF‐α and IL‐1β appeared significantly higher in the CLP group (*p < 0.05, ***p < 0.001 vs. Sham group), and the expression of TNF‐α and IL‐1β was reduced in the hippocampal tissues of mice in the CLP + OXA group compared with that of the CLP group (***p < 0.05, ***p < 0.001). These results suggested that OXA inhibits the production of inflammation after SAE.

### 3.5 OXA Inhibits Activation and Co‐Activation of Microglia and Astrocytes in the Hippocampus of SAE Mice

**FIGURE 5:**
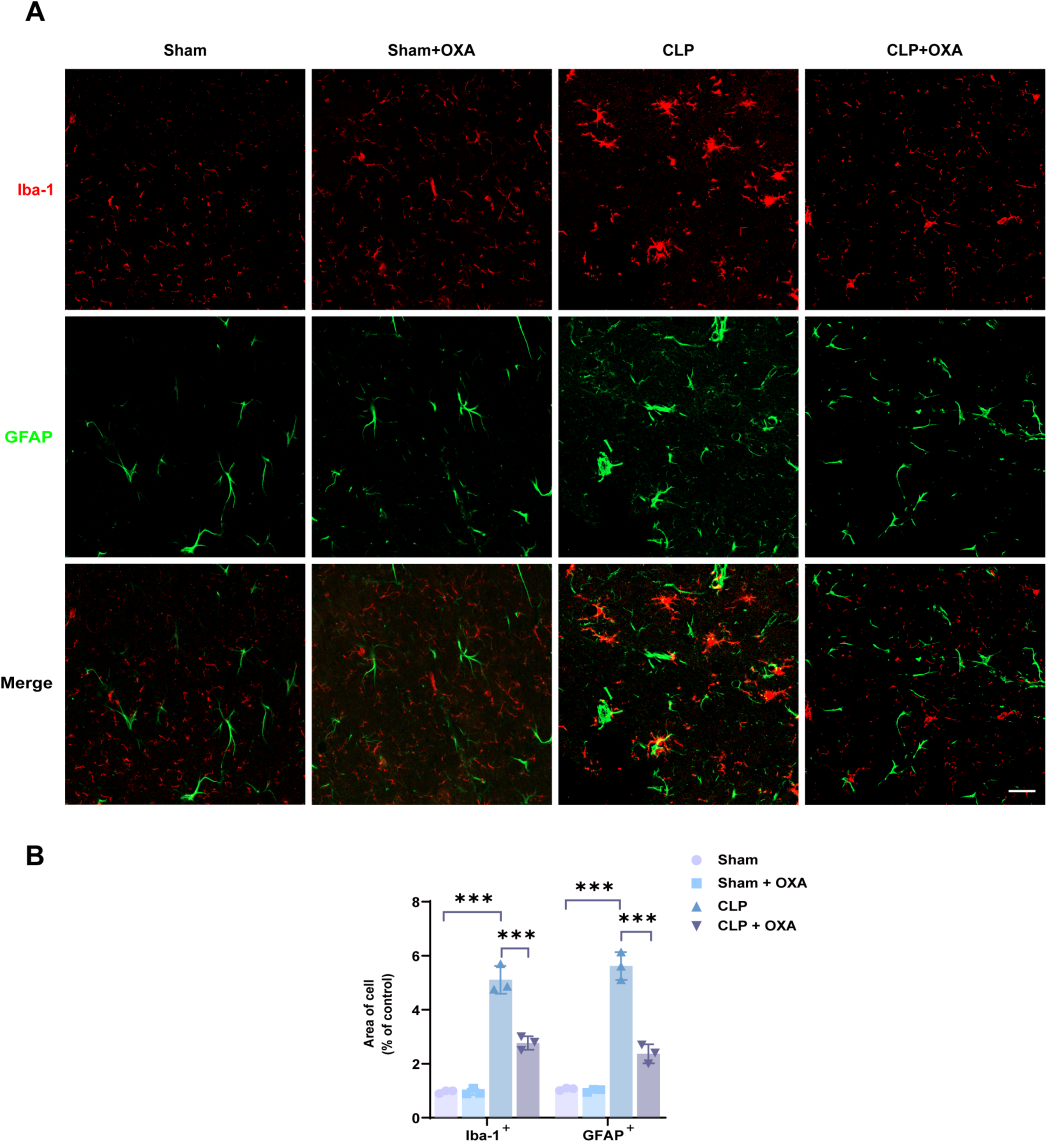
OXA attenuates activation and co‐activation of microglia and astrocytes in the hippocampus. (A) Representative immunofluorescence images showing the morphology of Iba1‐labeled microglia (red) and GFAP‐labeled astrocytes (green) in the hippocampus 7 days after the model. Simultaneous activation of Iba1‐labeled microglia and GFAP‐labeled astrocytes was close in expression, and OXA treatment inhibited the co‐activation of microglia and astrocytes. Scale bar = 20 μm. (B) Mean area of Iba‐1‐labeled single microglia and GFAP‐labeled single astrocyte in the hippocampal region. One‐way ANOVA and Tukey's post hoc test were used. The values were presented as mean ± SD (***p < 0.001, *n* = 3 for each group).

Microglial activation is a hallmark of SAE inflammation [3], and consistent with this, we observed that astrocytes were also activated after SAE. As shown in Figure 5, astrocyte (GFAP) and microglial (Iba‐1) positivity was more active in the CLP group compared to the Sham group. This was reflected in the enlarged cell bodies, shorter processes, and rounded or rod‐shaped cell morphology of the activated cells (***p < 0.001, ***p < 0.001 vs. Sham group). However, OXA treatment significantly attenuated CLP‐induced activation of microglia and astrocytes (***p < 0.001, ***p < 0.001 vs. CLP group). In addition, we found that activated microglia and activated astrocytes were close together in the CLP and CLP + OXA groups, suggesting that there may be co‐activation of microglia and astrocytes after CLP.

### 3.6 OXA Inhibits ERK/NF‐κB‐Mediated Activation of Microglia and Astrocytes After CLP

**FIGURE 6:**
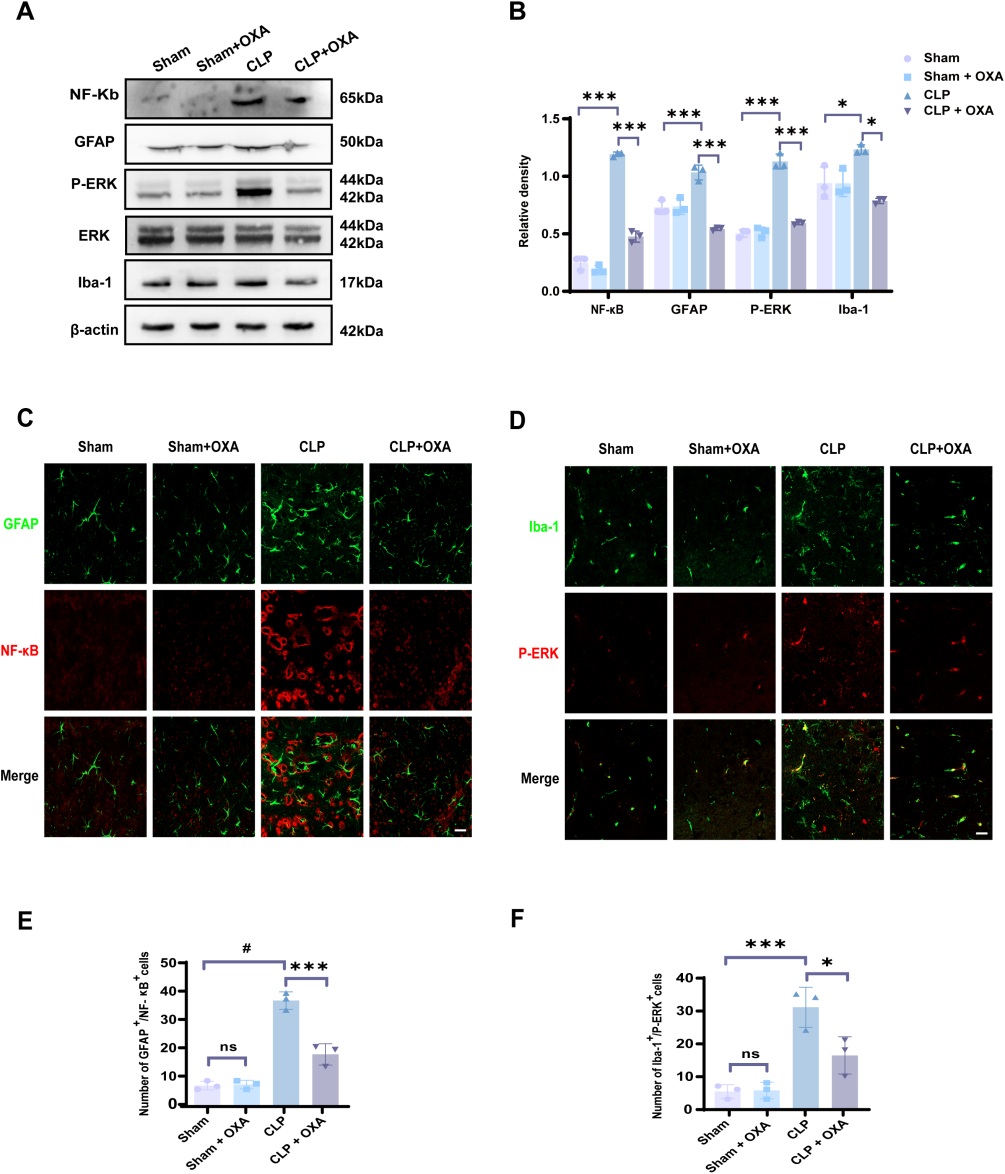
OXA reduces P‐ERK/NF‐κb‐mediated activation of microglia and astrocytes. (A, B) Protein expression and relative quantification of NF‐κB, P‐ERK, Iba‐1, and GFAP in hippocampal tissue 7 days after the model. (C) Representative immunofluorescence images of NF‐κB (red) and GFAP (green) co‐localization in the hippocampus 7 days after CLP. Scale bar = 20 μm. (D) Representative immunofluorescence images showing co‐localization of P‐ERK (red) and Iba‐1 (green) in the hippocampal region. Scale bar = 20 μm. (E) Percentage of NF‐κB positive cells that are GFAP positive in the hippocampus. (F) Percentage of P‐ERK‐positive cells that are Iba‐1‐positive in the hippocampus. One‐way ANOVA and Tukey's post hoc test were used. The values were presented as mean ± SD (*p < 0.05, ***p < 0.001, ^#^ *p* < 0.0001, *n* = 3 for each group).

We examined the protein expression levels of Iba‐1 and GFAP in hippocampal tissues 7 days after the model by Western blot, reflecting the activation of microglia and astrocytes, respectively. As shown in Figure 6A,B, the protein levels of GFAP and Iba‐1 were significantly higher in the CLP group (**p < 0.01, *p < 0.05 vs. Sham group), and the expression of GFAP and Iba‐1 was significantly lower in the CLP + OXA group compared to the CLP group (***p < 0.001, *p < 0.05). This is consistent with the results of the fluorescence. Our results confirmed the activation of microglia and astrocytes in the hippocampal region after the onset of SAE, and OXA inhibited the activation.

Microglia and astrocytes mediate inflammation, and the above results prompted us to further investigate the specific mechanisms of activation in microglia and astrocytes. We measured the expression levels of NF‐κB and phosphorylated ERK (P‐ERK) in the hippocampus. As shown in Figure 6A,B, the protein levels of P‐ERK and NF‐κB were significantly higher in the CLP group (***p < 0.001, ***p < 0.001 vs. Sham group), and the expression of P‐ERK and NF‐κB was significantly inhibited by OXA compared to the CLP group (***p < 0.001, ***p < 0.001).

To further validate that ERK and NF‐κB activated microglia and astrocytes, respectively, we investigated the co‐localization of GFAP and NF‐κB as well as Iba‐1 and P‐ERK in the hippocampal region. As Figure 6C,E shows, astrocytes mainly expressed NF‐κB, and there was no significant difference between the Sham group and the Sham + OXA group in terms of low expression of GFAP and NF‐κB. The CLP group showed significantly higher expression of GFAP and NF‐κB than that of the Sham group (^#^ *p* < 0.0001), and the OXA group significantly inhibited the expression of GFAP and NF‐κB (^****^ *p* < 0.001 vs. CLP group). Similarly, as shown in Figure 6D,F, P‐ERK were mainly expressed by microglia, and there was no significant difference in the expression of Iba1 and P‐ERK between the Sham group and the Sham + OXA group. The expression of Iba1 and P‐ERK was significantly elevated in the CLP group (***p < 0.001 vs. Sham group), and compared to the CLP group, the OXA treatment significantly inhibited the expression of Iba1 and P‐ERK (*p < 0.05).

### 3.7 OXA Administration Inhibits Hippocampal Type A1 Astrocytes and Promotes Type A2 Astrocytes After CLP

**FIGURE 7:**
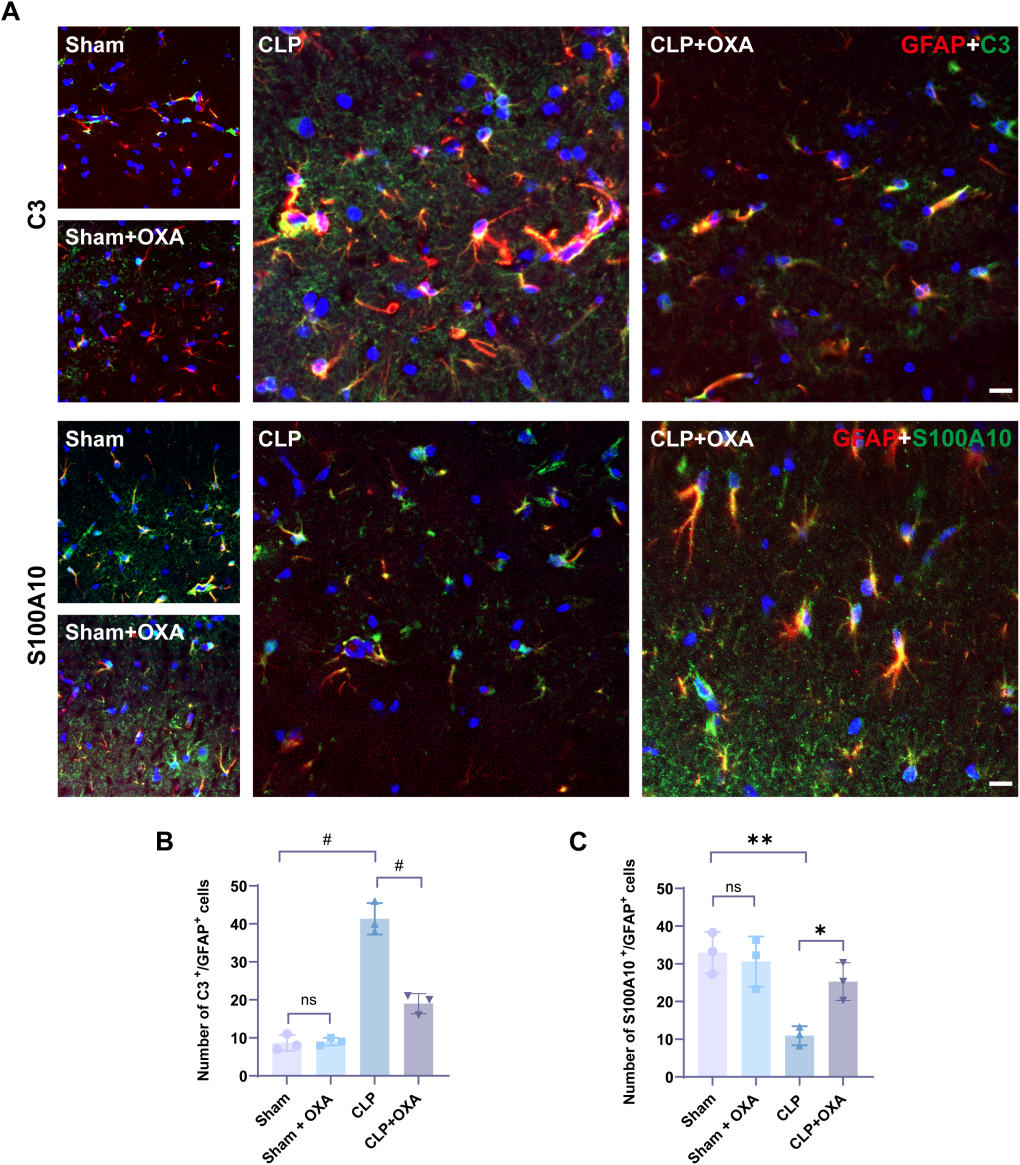
OXA affects the activation of hippocampal type A1/A2 reactive astrocytes. OXA treatment decreases the number of type A1 astrocytes and increases the number of type A2 astrocytes. (A) Representative immunofluorescence images showing the co‐localization of GFAP‐labeled astrocytes with c3‐labeled A1 astrocytes and the co‐localization of GFAP‐labeled astrocytes with S100A10‐labeled A2 astrocytes co‐localized in the hippocampus. Scale bar = 20 μm. (B) Percentage of c3‐positive cells in the hippocampus that are GFAP‐positive. (C) Percentage of S100A10 positive cells in the hippocampal region that are GFAP positive. One‐way ANOVA and Tukey's post hoc test were used. The values were presented as mean ± SD (*p < 0.05, **p < 0.01, ^#^ *p* < 0.0001, *n* = 3 for each group).

We examined four groups of hippocampal type A1 astrocytes (C3/GFAP‐labeled) and type A2 astrocytes (S100A10/GFAP‐labeled) and assessed the effect on the number of hippocampal C3/GFAP‐positive cells and S100A10/GFAP‐positive cells after OXA treatment using immunofluorescence staining. As shown in Figure 7A, there was no significant difference in the number of C3/GFAP‐positive cells and S100A10/GFAP‐positive cells between the Sham group and the Sham + OXA group. There was a significant increase in the number of C3/GFAP‐positive cells and a significant decrease in the number of S100A10/GFAP‐positive cells in the CLP group compared to the Sham group (#*p* < 0.0001, **p < 0.01). The number of C3/GFAP‐positive cells in OXA‐treated CLP mice was significantly lower than in the CLP group (#*p* < 0.0001), and the number of S100A10/GFAP‐positive cells was significantly higher than in the CLP group (*p < 0.05).

## 4 Discussion

Sepsis usually causes acute or long‐term cognitive and affective deficits and leads to death and poor prognosis in septic patients [28]. The hippocampus is known to be a key brain region involved in learned behavior and emotional orientation. Lesions of the hippocampal region can cause severe cognitive deficits and affect the brain's ability to perform functions such as memory [29, 30, 31]. By assessing the emotional and cognitive functions of animals, including OFT, Y‐maze, and behavioral experiments with TST, we found that post‐sepsis mice showed a significant decrease in exploratory behavior and an apparent anxiety‐like state. Our findings also revealed that mitochondrial structures in hippocampal tissue showed significant swelling and absence of cristae after CLP. Studies have confirmed that mitochondrial function is impaired during sepsis [32]. After mitochondrial damage, the normal physiological functions of neurons could not be met, which led to neuronal apoptosis [33], and apoptosis was involved in the development of SAE [8, 34]. This shows the important role of the disruption of redox homeostasis in the pathogenesis of sepsis‐associated encephalopathy [35]. In our experiments, we observed apoptosis and survival of hippocampal neurons by TUNEL and NeuN staining. The results of section staining showed that the number of damaged hippocampal neurons was significantly increased in septic mice. This also confirms that one of the main core and pathological bases for the development of sepsis‐induced cognitive impairment may be the impairment of the antioxidant defense system.

Under normal conditions, intracellular ROS can act as intracellular signaling molecules to regulate physiological functions of the organism, but excessive accumulation of ROS is able to cleave IL‐1β precursors into mature IL‐1β and mediate inflammation [36]. Controlling ROS production to inhibit oxidative stress‐induced inflammatory damage has been reported in a variety of diseases [37, 38], and interventions targeting oxidative stress in the hippocampus of the brain in an experimental model of sepsis can show promise for improving cognitive impairment [39]. Previous studies have suggested that OXA reduces oxidative stress damage in acute kidney injury [40] and rheumatoid arthritis [41] by downregulating ROS. In the present study, we examined the changes in ROS content in the hippocampal region of the brain and the effect of OXA on it in CLP‐induced SAE mice. Recent studies have pointed out that intranasal administration of peptides for the treatment of cognitive impairment disorders demonstrates promising therapeutic approaches [21, 42]. Transnasal drug delivery has clear advantages. Appetin A delivered transnasally has been shown to target the central nervous system, with the highest concentrations observed in the trigeminal nerve, olfactory bulb, and anterior olfactory nucleus, this method also significantly reduces delivery to the blood and peripheral tissues compared to intravenous (IV) administration [43]. Thus, transnasal orexin may be a promising noninvasive treatment for cognitive impairment. However, few studies have investigated the effects of orexin ingestion on cognitive aspects. In order to observe the effects of OXA septic brain injury on cognitive impairment, we observed the effects of OXA septic brain injury on cognitive impairment after 7 consecutive days of administration. Our results showed that ROS expression was significantly enhanced in the hippocampal tissue of mice after CLP‐induced brain injury, and interestingly, intranasal administration of OXA significantly reduced the elevation of ROS in the hippocampal region. This suggests an ameliorative effect of OXA on oxidative stress in post‐septic neurons of mice. This should be the first exploration of OXA against CLP‐induced SAE in terms of oxidative stress injury. In addition, we sought to assess the mechanism by which OXA protects against CLP‐induced oxidative stress. The NOX family of NADPH oxidases has the ability to produce superoxide and downstream ROS, and NOX2 appears to be most widely distributed among the NOX iso forms [44]. It was also noted that NOX2 is localized to synaptic sites in hippocampal neurons, which may play a role in superoxide‐dependent long‐duration enhancement and memory function [45]. Based on this, we examined the expression of NOX2 in each group to explore the effect of OXA on it. Our results showed that NOX2 expression was enhanced in the hippocampal tissues of mice in the CLP group, and OXA decreases CLP‐induced levels of NOX2 and ROS in the mouse hippocampus, suggesting that CLP‐induced oxidative stress may be caused by an increase in oxidative stress through an increase in NOX2 expression. In other words, OXA reversed CLP‐induced oxidative damage by regulating NOX2 expression.

Recent studies have indicated that increasing ROS concentration promotes microglia activation [46]. Activated microglia and astrocytes release inflammatory cytokines and chemokines that induce mitochondrial dysfunction and neuronal damage, exacerbating the inflammatory environment in the brain [47]. In contrast, astrocytes shift to different activation states under different brain disease conditions. One is the neurotoxic type A1, which releases neurotoxic complement 3 (C3) from type A1 astrocytes, leading to neuronal death [48]. The second is the neuroprotective type A2, which promotes neuronal survival and tissue repair [49] and is labeled with S100A10. In models of cerebral ischemia, astrocytes exhibit a neuroprotective subtype, whereas under neuroinflammatory conditions, inflammatory microglia are able to induce astrocyte activation into a neurotoxic subtype by secreting three factors, IL‐1β, TNF‐α, and C1q. The immune response of astrocytes, at least under inflammatory conditions, is dependent on microglia [50]. In the present study, we assessed the status of microglia and astrocytes after CLP and the effect of OXA on the mediated neuroinflammatory process. We observed hypertrophied cell bodies, shorter protrusions, and an increased number of activated cells, which could be seen in microglia with an “amoeboid” appearance and in astrocytes with enlarged rod‐like bodies, suggesting a significant activation of both. At the same time, fluorescence immunostaining revealed a close co‐activation of microglia and astrocytes. Co‐activation of microglia and astrocytes releases large amounts of inflammatory cytokines, leading to a severe inflammatory response and aggravated brain damage [51]. Further, we found that serum and hippocampal tissue IL‐1β and TNF‐α expression levels were significantly increased in SAE and that OXA treatment significantly inhibited hyperactivation of microglia and A1‐type astrocytes after CLP injury, increased A2‐type astrocyte activation, and significantly decreased pro‐inflammatory cytokines. However, the underlying mechanisms are unclear.

ERK is a mitogen‐activated protein kinase (MAPK) and treatments targeting ERK overactivity in microglia have shown promise in CNS disorders [52, 53]. In addition, studies have confirmed that NF‐κB regulates the expression of several inflammatory cytokines involved in the inflammatory process of CLP [54, 55]. Activation of NF‐κB significantly promotes the expression and secretion of inflammation in astrocytes [56], whereas inhibition of NF‐κB signaling pathway activity prevents SAE, exerts neuroprotective functions [57, 58], and ameliorates neuronal apoptosis [59]. In further exploring oxidative stress‐induced inflammation and its key signaling mechanisms, we found that microglia and astrocytes activated ERK and NF‐κB pathways, respectively, after CLP injury occurred. Furthermore, OXA treatment significantly inhibited the expression of P‐ERK and NF‐κB in the hippocampus. Thus, our results demonstrate that the anti‐inflammatory effect of OXA in CLP‐induced brain injury may be through the inhibition of ERK/NF‐κB signaling pathway activation in microglia and astrocytes.

## 5 Conclusions

In conclusion, our results suggest that OXA inhibits the development of oxidative stress by decreasing NOX2 in the hippocampus, reducing ROS content, and attenuating mitochondrial damage, thereby reducing the expression of pro‐inflammatory cytokines IL‐1β and TNF‐α, and the main mechanism of OXA may be through inhibiting the activation of the ERK/NF‐κB signaling pathway in microglial cells and A1‐type astrocytes. Our findings suggest that OXA may be a potential therapeutic agent for infectious brain injury associated with inflammation and oxidative stress, providing a new therapeutic strategy for OXA treatment of septic brain injury.

## Author Contributions

J.G. conceived and designed the study; J.G., D.K., J.L., and S.Y. established the animal models; T.X., F.W., J.H.L., and C.F.C. performed the behavioral tests; J.D., M.D., Z.K., and G.H. analyzed and interpreted the data; J.G. wrote and prepared the manuscript; J.L., Y.T., and J.Z. reviewed the manuscript; J.Z. coordinated the study. All authors have read and approved the final manuscript.

## Conflicts of Interest

All the authors declared no conflicts of interest.

## Supporting information


Supporting Information S1.

## Data Availability

All relevant data are included in the manuscript and Supporting Information S1. Further inquiries can be directed to the corresponding authors.
