# Supplementary material for: Orexin‐A Attenuates the Inflammatory Response in Sepsis‐Associated Encephalopathy by Modulating Oxidative Stress and Inhibiting the ERK/NF‐κB Signaling Pathway in Microglia and Astrocytes
Source: CNS Neurosci Ther. 2024 Nov 7;30(11):e70096. doi: 10.1111/cns.70096 (PMC11541240; doi:10.1111/cns.70096)

Full unedited gel/blot for Figure. 3D

NOX2 55 KDa

Repeat 1

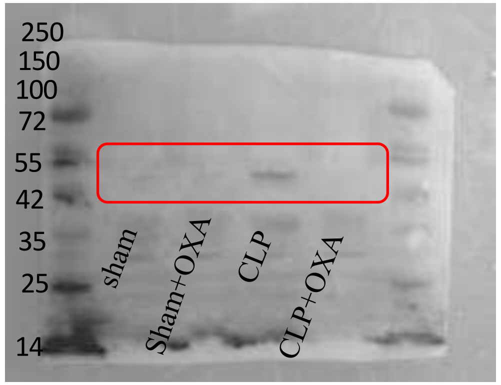

Repeat 2

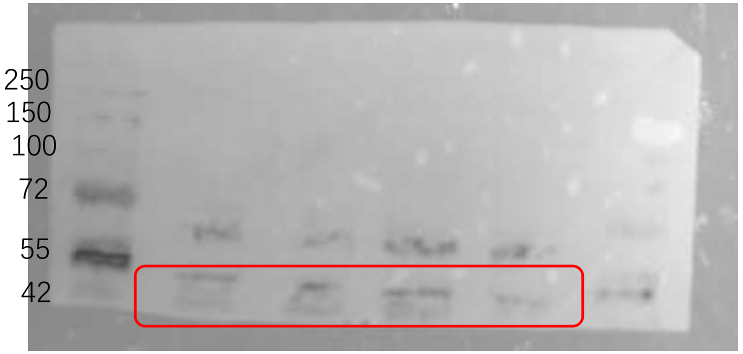

Repeat 3

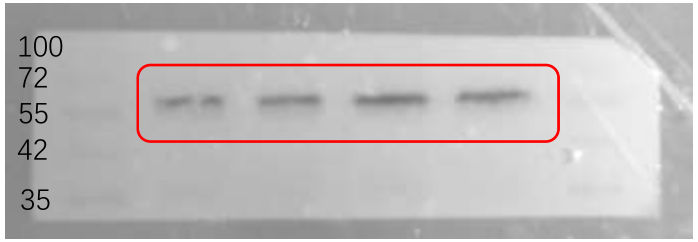

β-actin 42 KDa

Repeat 1

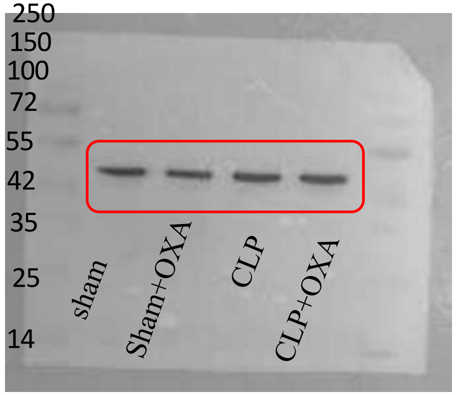

Repeat 2

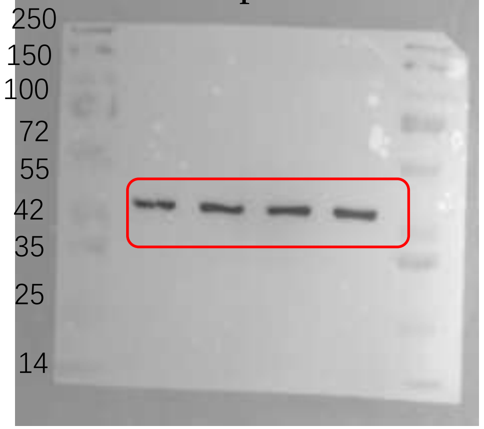

Repeat 3

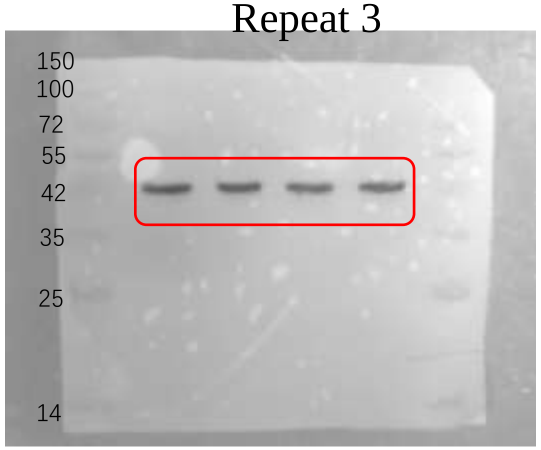

Full unedited gel/blot for Figure. 4C

IL-1β 35 KDa

Repeat 1

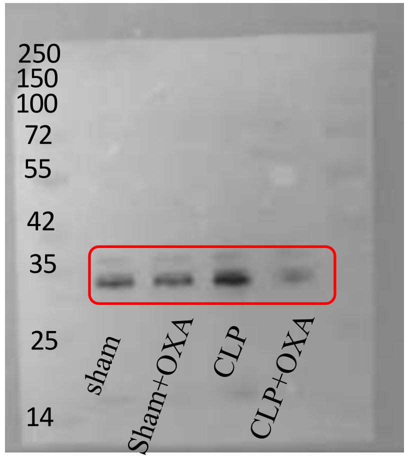

Repeat 2

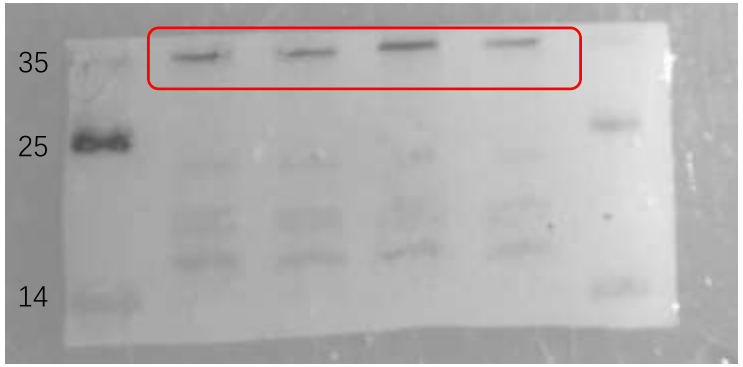

Repeat 3

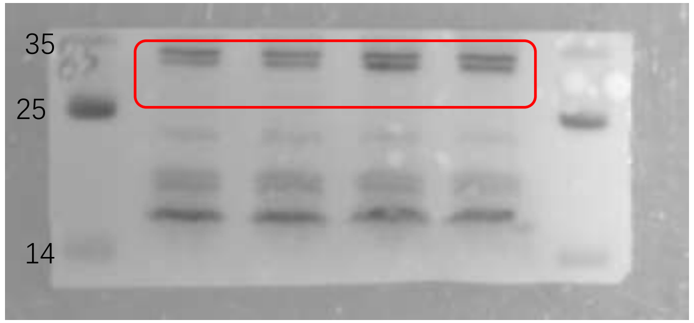

TNF- a 17KDa

Repeat 1

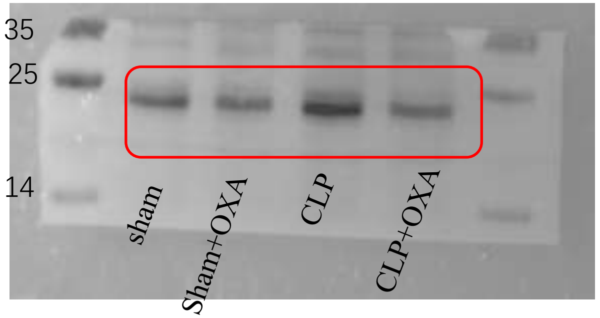

Repeat 2

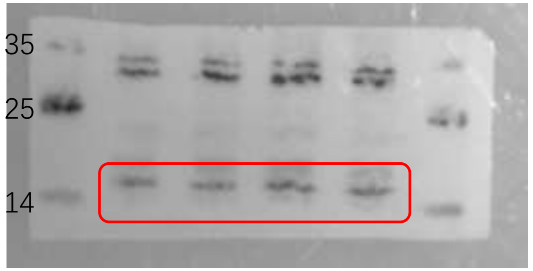

Repeat 3

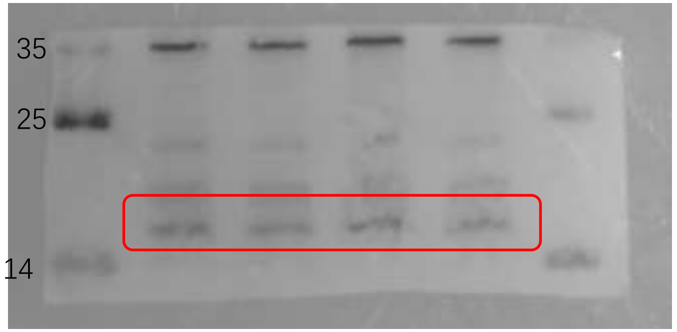

Full unedited gel/blot for Figure. 6A

ERK 42/44KDa

Repeat 1

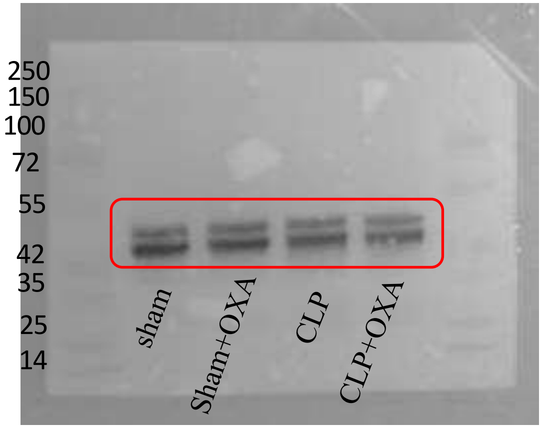

Repeat 2

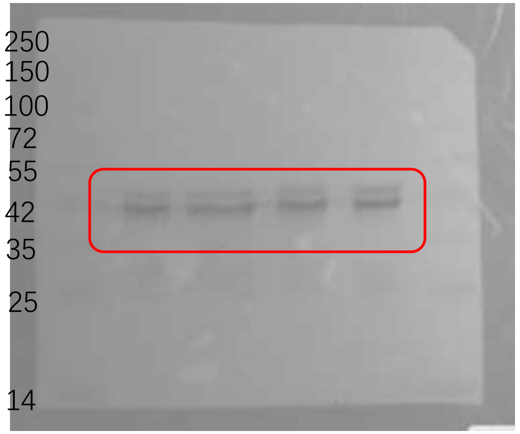

Repeat 3

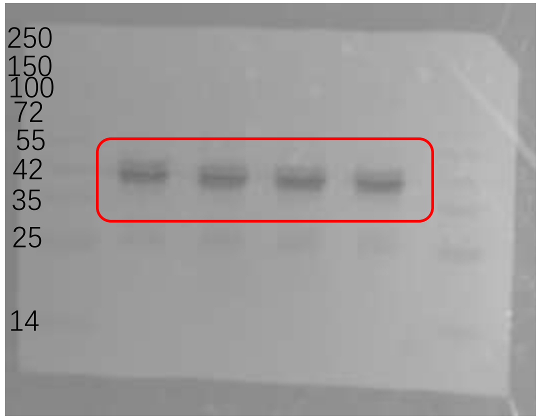

P-ERK 42/44 KDa

Repeat 1

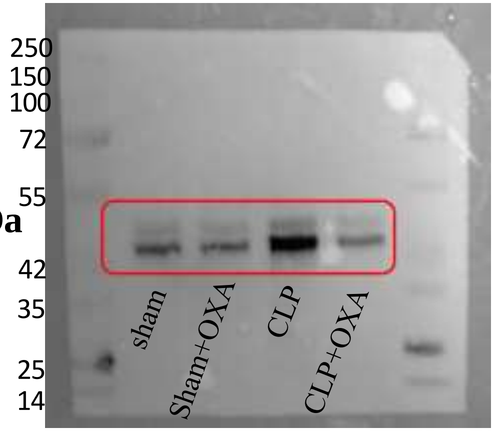

Repeat 2

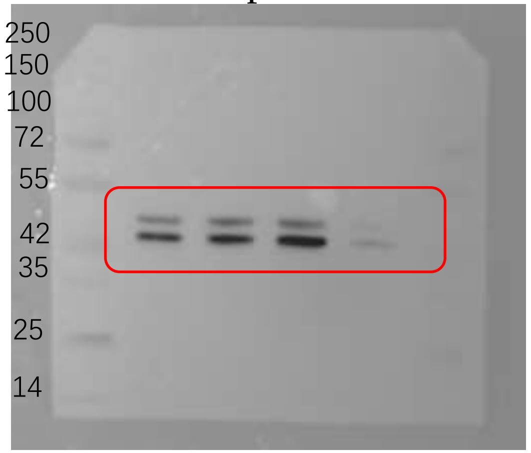

Repeat 3

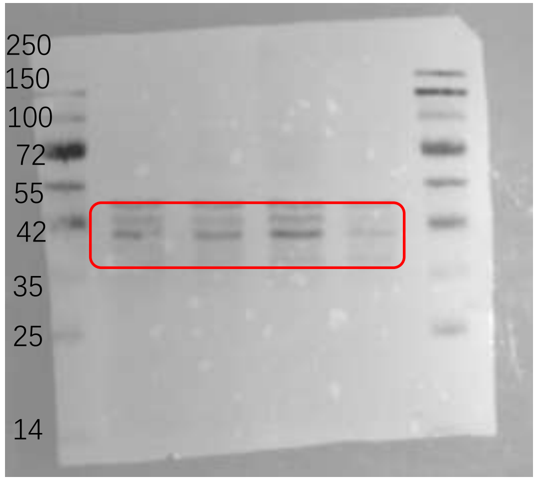

Full unedited gel/blot for Figure. 6A

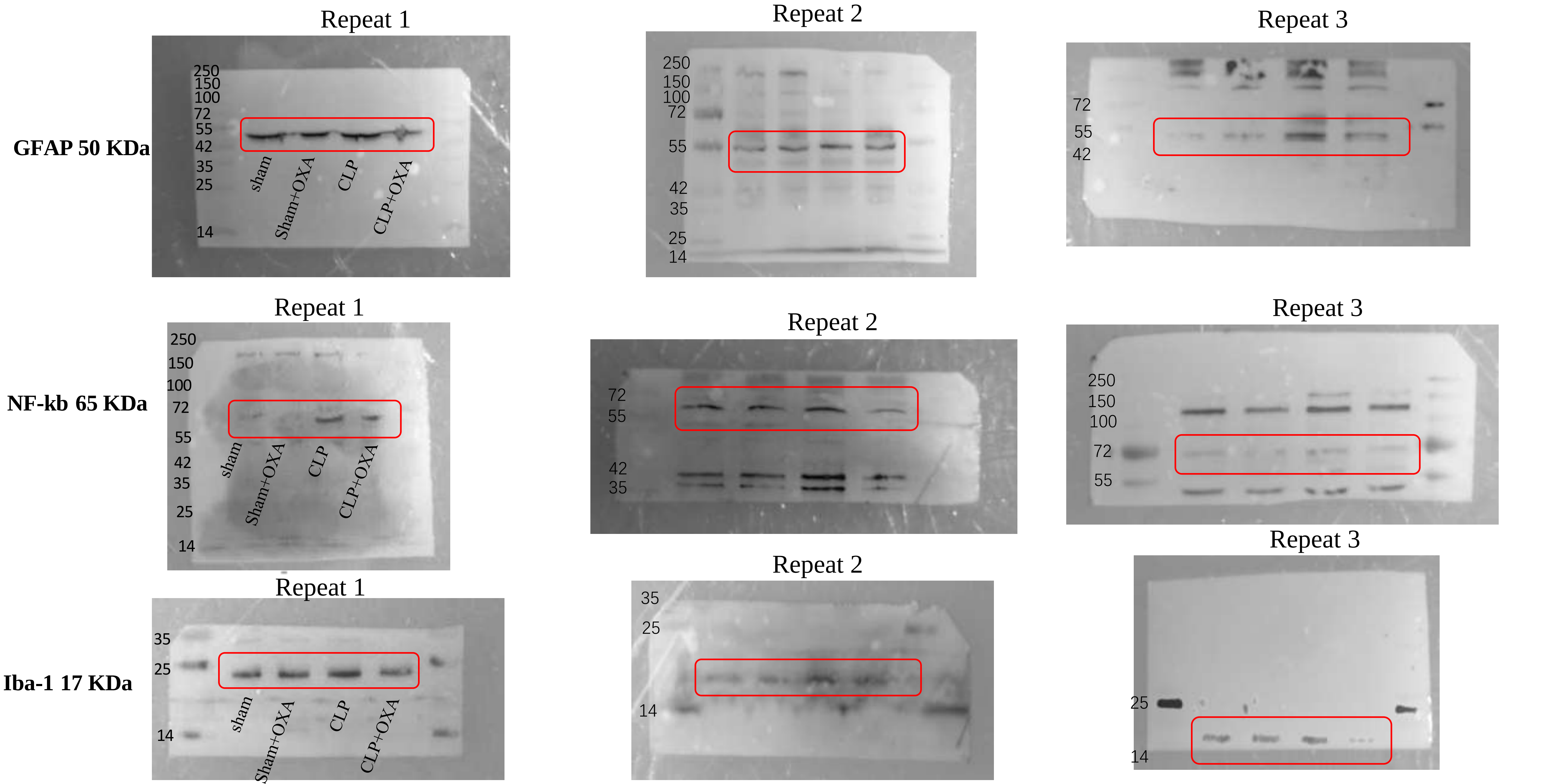

Supplement: Supplementary file 1 — Supporting Information S1. [file CNS-30-e70096-s001.pdf]
